# Burden and Determinants of Chronic Kidney Disease Among Diabetic Patients in Ethiopia: A Systematic Review and Meta-Analysis

**DOI:** 10.3389/phrs.2021.1603969

**Published:** 2021-04-09

**Authors:** Tadesse Tolossa, Getahun Fetensa, Bikila Regassa, Mekdes Tigistu Yilma, Merga Besho, Ginenus Fekadu, Bizuneh Wakuma, Daniel Bekele, Diriba Mulisa

**Affiliations:** ^1^ Department of Public Health, Institute of Health Science, Wollega University, Nekemte, Ethiopia; ^2^ Department of Nursing, Institute of Health Science, Wollega University, Nekemte, Ethiopia; ^3^ Department of Midwifery, Institute of Health Science, Wollega University, Nekemte, Ethiopia; ^4^ Department of Pharmacy, Institute of Health Science, Wollega University, Nekemte, Ethiopia; ^5^ School of Pharmacy, Faculty of Medicine, The Chinese University of Hong Kong, Shatin, NT, Hong Kong; ^6^ Department of Statistics, College of Computational Science, Dire Dawa University, Dire Dawa, Ethiopia

**Keywords:** kidney disease, nephropathy, diabetic, systematic review, Ethiopia

## Abstract

**Background:** Chronic kidney disease (CKD) among diabetic patients is becoming a global health burden with a high economic cost to health systems. The incidence of CKD is higher in low-income countries such as Ethiopia. In Ethiopia, there is no national representative evidence on the burden and determinants of CKD among patients with diabetes. Therefore, this review aimed to estimates the pooled burden and determinants of CKD among patients with diabetes.

**Methods:** Published articles from various electronic databases such as Pub Med, Google Scholar, CINAHL, Scopes, Cochrane library, the Web of Science, and African Journals Online were accessed. Also, unpublished studies from Addis Ababa digital library were identified. We included all observational studies (cross-sectional, case-control, and cohort) in the review. Data were extracted on the Microsoft Excel spreadsheet and analyzed using STATA 14.1 version. A random-effects model was used to estimate the pooled estimate with a 95% confidence interval (CI). Forest plots were used to visualize the presence of heterogeneity and estimate the pooled burden and determinants of chronic kidney disease among diabetic patients. The presence of publication bias was assessed by funnel plots and Egger’s statistical tests.

**Results:** Published (297) and unpublished (2) literature were identified from several databases and digital libraries, of which twelve articles were selected for final meta-analysis. Significant heterogeneity was observed across studies (I^2^ = 85.2%), which suggests a random-effects model to estimate pooled burden. The analysis found that the pooled burden of CKD among patients with diabetes was 18.22% (95% CI: 15.07–21.38). Factors such as hypertension (OR = 2.65, 95%, CI: 1.38, 5.09), type of DM (OR = 0.33, 95%, CI: 0.14–0.76), and duration of DM (OR = 0.51, 95%, CI: 0.34–0.77) were found to have significant association with CKD.

**Conclusion:** The current review revealed a higher burden of CKD among patients with diabetes in Ethiopia. The presence of hypertension, type II diabetes, and duration of diabetes for a longer duration were found to be independent determinants of CKD among patients with diabetes. For better control of chronic kidney disease, integrated management of hypertension and DM should be designed with a special focus on chronic diabetic patients.

## Background

Chronic kidney disease is a state in which the kidneys cannot filter blood to the body as a normal kidney, and which results in the accumulation of excess fluid and waste in the body [1]. CKD is a global health burden with a high economic cost to health systems [2]. Worldwide, in 2016 there were more than 276 million prevalent cases and 21 million new cases of CKD [3].

Literature has shown that the magnitude of CKD varies from country to country, but it is more frequent in African-Americans, Asian-Americans, and Native Americans [4]. Around 63% of chronic kidney disease is found in low and lower-middle-income countries [3]. A study conducted among American Indians and Alaska Natives revealed the magnitude of CKD was 29% [5]. The prevalence of CKD was 30–60% in South Asia [6]. Another study conducted in China shows the prevalence of CKD among patients diagnosed with diabetes was 30.9% [7].

Studies from SSA countries also indicated the increasing burden of CKD among diabetic patients. Shortage of adequate funds and trained human resources as there is continuing ‘brain drain’ of health care workers from Africa to more prosperous countries of the world, lack of regional registries were among factors for the increasing burden of the disease [8, 9]. A systematic review and meta-analysis from 98 studies conducted in Africa showed the prevalence of CKD was 15.8% [10]. In Ethiopia, the prevalence of CKD ranges from 14.3 [11] to 26.3% [11].

Currently, most developing countries are experiencing rapid epidemiological transitions and are provoked with the double burden of communicable and non-communicable diseases [12]. This dual burden has led to a consequential rise in the number of people affected by CKD in the developing countries, especially sub SSA [3, 13].

Over the last decades, diabetes followed by hypertension is considered the two prominent drivers of CKD [3, 14]. Diabetic nephropathy is one of the most common complications of diabetes. Globally, diabetes mellitus accounts for 50.6% of the overall increase in CKD disability-adjusted life-years (DALYs). The increase in the burden of CKD due to diabetes and elevated blood pressure happened at a much faster rate in developing countries than in high-income countries [3]. A systematic review conducted in SSA showed that 95% of diabetic patients develop kidney disease after 10 years from diabetes diagnosis, an estimated 35% may develop the end-stage renal disease after 5 years and 18% die from kidney disease after 20 years of diabetic diagnosis [14].

CKD results in increased premature mortality, development of the end-stage renal disease, cardiovascular diseases, and rising health-care costs [14]. For examples, with large numbers of end-stage renal disease patients in SSA, treatment, and care for chronic kidney disease is very challenging due to inadequate facilities and lack of funding for dialysis [8].

Globally, factors such as an increase in age [14–16], type II DM [16], hypertensive patients [14, 16–18], poor knowledge about the disease [17], living with diabetes for longer duration [14, 16, 17], increased BMI [16] and obesity [14] are important contributing factors for the occurrence of CKD among patients with diabetes.

Blood sugar controls are very important for delaying the onset of diabetic complications specifically for patients with CKD. Delaying the occurrence of complications requires knowledge of how kidney disease will affect metabolism and how medication can be safely used [19]. The management of diabetes is predicated on the basis of reducing hyperglycemia to improve hyperglycemic symptoms, will prevent the onset, and slow down progression of renal complications over time [20]. In another way, the longer duration of living with DM, the probability of having kidney disease will be increased [21]. Contrarily in developing countries like Ethiopia, this reality are not well addressed and that is why this review and meta-analysis are required. In addition, there is no nationally representative data on the burden of CKD among patients with diabetes in Ethiopia. Therefore, this systematic review and meta-analysis were aimed to estimate the pooled burden of chronic kidney disease and its determinants among diabetic patients in Ethiopia.

Assessing the pooled burden and determinants of CKD led us to develop and design intervention which used to reduce the burden of the disease, particularly in resource-limited settings such as Ethiopia. The implications of the findings of our study are for national policymakers, program managers, and non-governmental organizations to reduce the burden of CKD among patients with diabetes in low-resource settings by developing appropriate interventions.

## Methods

### Search Strategy and Review Process

The Preferred Reporting Items for Systematic Reviews and Meta-Analyses (PRISMA) checklist was used to report the review. Endnote version X7.2 was used to maintain and manage the citation and facilitate the review process (Supplementary File S1) [22]. In the initial step of the search process, we checked for the presence of the existing systematic review and meta-analysis on a similar topic by extensive search of literature. All pertinent published studies in the following major databases; PubMed, Google Scholar, CINAHL, Scopes, Cochrane library, the Web of Science, and African Journals Online were involved in the review. The reference lists of identified studies were also reviewed to find additional articles. Unpublished studies were retrieved from the official website of the Addis Ababa University electronic database. Pre-identified search terms were used to allow a comprehensive search strategy that included all the relevant studies. The search terms such as “chronic kidney disease, renal failure, nephropathy, diabetes mellitus, diabetes complications, chronic complication of DM, diabetic patients, and Ethiopia” separately and/or in combination were used.

#### Eligibility Criteria

##### Inclusion Criteria

**Participants:** All diabetic patients regardless of age and types of DM.

**Study design:** All observational study designs (cross-sectional, case-control, and cohort) were included.

**Setting:** Studies only conducted in Ethiopia.

**Study:** All studies (published and unpublished) that were published in the form of journal articles, master’s thesis, and dissertation till the final date of data analysis were included.

**Language:** Only English language was considered in this study.

##### Exclusion Criteria

We excluded articles that were not fully accessible, after at least two-email contact with the principal authors.

### Measurement of Outcome Variables

The burden of CKD among patients with diabetes was the first outcome of the study. It is measured as the total number of CKD cases over a total number of all diabetic patients multiplied by 100. CKD was defined using glomerular filtration rate (eGFR) < 60 ml/min/1.73 m^2^) and the presence of albuminuria [16]. The glomerular filtration rate (GFR) was estimated using the modification of diet in renal disease (MDRD) and Cockcroft Gault equations [16]. A determinant of CKD among patients with diabetes was the second outcome of this study. For binary data (determinants of CKD), the input variables required by “metan” should contain the cells of the 2 *×* 2 table; i.e., the number of individuals who did and did not experience the CKD in the exposed and non-exposed groups for each study. All potential determinants related to CKD were determined using the odds ratio (OR) and calculated based on binary outcomes from the included primary studies. Sex of patients (male vs. female), hypertension (presence of hypertension vs. absence of hypertension), types of diabetes (type I vs. type II), family history of CKD (presence vs. absence), current alcohol consumption (no vs. yes), duration of diabetes (<10 years vs. ≥10 years), and BMI (<24.5 Kg/m^2^ vs. ≥24.5 Kg/m^2^) were potential variables which selected in the analysis.

### Quality Assessment and Data Abstraction

To assess the quality of the data, the Joanna Briggs Institute Meta-Analysis of Statistics Assessment and Review Instrument (JBI-MAStARI) was used [22]. Two reviewers (TT and BR) independently extracted the data using a standardized data extraction checklist on a Microsoft excel spreadsheet. In the beginning, articles were downloaded and supplemented to Endnotes version 7.2 reference management. Duplicated articles were excluded by using Endnotes reference management. Then, the titles and abstracts of the studies were exhaustively assessed based on the relevance of the outcome. The full-text of the remaining articles was evaluated for eligibility based on prearranged inclusion and exclusion criteria. For the first outcome variable (burden of CKD among patients with diabetes), the checklist for data extraction contains the title, author name, year of publication, region (the area where the study was conducted), study design, outcome measurements, sample size, response rate and a number of participants with cases (Table 1). After two data extractors performed the review, any discrepancy was resolved by including the third reviewer (MT) for a possible consensus. When articles did not have adequate data, corresponding authors of the research articles were contacted through their email.

**TABLE 1:** Summary of included studies regarding burden and determinants of CKD among patients with diabetes in Ethiopia, 2020.

S.N	Author	Year	Region	Study design	Study area	Method of estimating eGFR	CKD definition	Sample size	Response rate (%)	No of CKD	Burden of CKD (95% CI)
1	Temesgen, et al. [16]	2014	SNNP	Cross-sectional	Butajira hospital	MDRD	eGFR <60 ml/min/1.73 m^2^	214	100	39	18.2 (13.0, 23.4)
2	Kidist et al. [15]	2017	Amhara	Cross-sectional	Felegehiwot hospital	Not stated	eGFR <60 ml/min/1.73 m^2^	344	100	39	11.3 (7.9, 14.6)
3	Kabaye et al. [17]	2019	Oromia	Cross-sectional	JUMC	MDRD	eGFR <60 ml/min/1.73 m^2^	208	100	54	25.9 (20.0, 31.9)
4	Shawaneh et al. [16]	2018	Amhara	Cross-sectional	UGH	MDRD	eGFR <60 ml/min/1.73 m^2^	229	100	50	21.8 (16.4, 27.1)
5	Dawit et al. [25]	2010	Oromia	Cross-sectional	JUMC	Not stated	eGFR <60 ml/min/1.73 m^2^	305	100	48	15.7 (11.6, 19.8)
6	Gizaw et al. [26]	2015	AA	Cross-sectional	Black lion hospital	Not stated	eGFR <60 ml/min/1.73 m^2^	418	100	39	9.3 (6.5, 12.1)
7	Getahun et al. [18]	2019	AA	Cross-sectional	Black lion hospital	MDRD	eGFR <60 ml/min/1.73 m^2^	163	100	39	23.9 (17.3, 30.4)
8	Meron [24]	2016	AA	Cross-sectional	AA	MDRD and cockcroft gault equation	eGFR <60 ml/min/1.73 m^2^	355	100	68	19.1 (15.0, 23.2)
9	Solomon et al. [28]	2017	Tigrai	Case control	Ayider referral hospital	Not stated	eGFR <60 ml/min/1.73 m^2^	420	100	84	20.0 (16.1, 23.8)
10	Alemayehu et al. [11]	2018	AA	Retrospective follow up	St. Paul’s hospital	Cockcroft-gault equation	eGFR <60 ml/min/1.73 m^2^	435	100	62	14.2 (10.9, 17.5)
11	Hailemaryam et al. [27]	2020	Amhara	Cross-sectional	UGH	MDRD	eGFR <60 ml/min/1.73 m^2^	272	100	47	17.3 (12.7, 21.7)
12	Temesgen F. [11]	2020	Amhara	Cross-sectional	Dessie referral hospiatl	MDRD	eGFR <60 ml/min/1.73 m^2^	323	100	83	25.7 (20.9, 30.4)

AA, addis ababa; CKD, chronic kidney disease; eGFR, glomular filtration rate; JUMC, jimma university medical college; MDRD, modification of diet in renal disease; SNNP, southern nation, nationalities and peoples; UGH, university of gondar hospital.

### Heterogeneity and Publication Bias

Cochran *Q* test (chi-squared statistic) and inverse variance (I^2^) test statistic on forest plot were used to check heterogeneity among the included studies. Cochran’s *Q* statistical heterogeneity test is considered statistically significant at *p* ≤ 0.05. For the first outcome, a high degree of heterogeneity was observed; hence, a random-effects model was used to estimate the pooled burden of chronic kidney disease among diabetic patients. For the second outcome, heterogeneity was not observed for three factors (sex of participants, duration of DM, and family history of kidney disease); hence, a fixed-effects model was computed. For the remaining three factors (hypertension, types of DM, and BMI) moderate to a high degree of heterogeneity was observed and random-effects model was used to estimate the Der Simonian and Laird’s pooled effect. To identify the source of heterogeneity, meta-regression was conducted using sample size and year of publication. In addition, subgroup analysis was performed using region where the studies were conducted and statistically significant results were declared in the presence of heterogeneity. The publication bias was checked by the funnel plot. In addition, Egger’s weighted regression and Begg’s test were used to check publication bias [23]. A *p*-value of less than 0.05 was used to declare the statistical significance of publication bias.

### Data Analysis

After important information was extracted on Microsoft excel from each original study, the data were exported to STATA for windows version 14 for analysis. The burden of CKD with 95% confidence interval and OR of the association between CKD among patients with diabetes and its determinants were presented in the form of a forest plot.

## Results

### Study Selection

**FIGURE 1:**
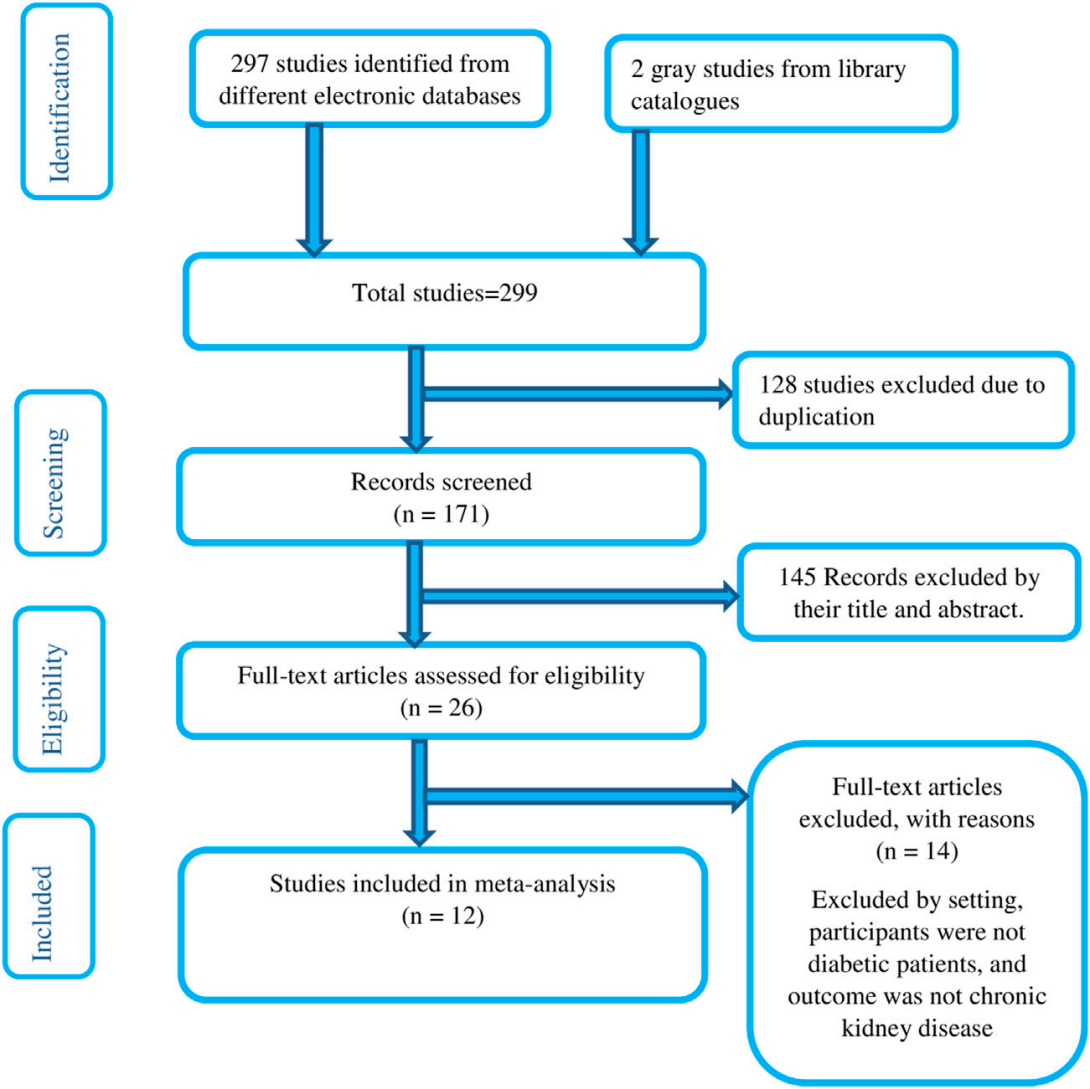
Flow diagram of the studies included in the meta-analysis.

A total of 297 published and two unpublished literatures were identified from several electronic databases and Addis Ababa digital library, respectively. Of the total identified studies, 128 duplicates papers were removed and 145 records were excluded by reviewing titles and abstracts. The full text of the remaining 26 studies was assessed and screened for eligibility. Accordingly, 14 studies were excluded based on pre-determined eligibility criteria. Finally, 12 articles fulfilled the eligibility criteria were included in the final analysis. We used the Preferred Reporting Items for Systematic Reviews and Meta-Analyses (PRISMA) flow diagram to present the systematic review overview (Figure 1).

### Features of Included Studies

Of the total included studies, all were published articles except one study which was retrieved from gray literature [24]. Regarding study design, ten studies were cross-sectional study design [11, 15–18, 24–27], one case-control [28], and one retrospective cohort study design [11]. A total of 3,686 patients with diabetes have participated in this study. The sample sizes range from a minimum of 163 [18] to a maximum of 435 [11]. Of the 12 studies included in the final analysis, four studies were conducted in Addis Ababa [11, 18, 24, 26], two in Oromia region [17, 25], four in the Amhara region [11, 15, 16, 27], one in SNNP region [16] and one in Tigrai region [28] (Table 1).

### Burden of Chronic Kidney Disease Among Patients With Diabetes in Ethiopia

**FIGURE 2:**
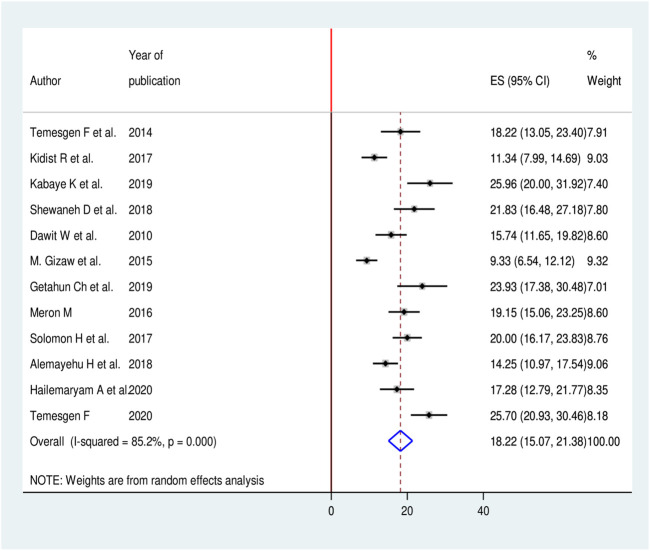
Forest plot for pooled burden of CKD among patients with diabetes in Ethiopia, 2020.

In this meta-analysis, we found significant heterogeneity across studies (I^2^ = 85.2%, *p* < 0.001), which is an indicator to use the random effects-model to estimate the pooled burden of CKD among patients with diabetes. The findings of original studies indicated there was an uneven and inconclusive burden of CKD among patients with diabetes in Ethiopia. From forest plot the largest burden was observed in a study conducted in Jimma University Medical Center (JUMC), Oromia region 25.9 (95% CI: 20.0, 31.9) [17] while the smallest burden was reported in Black Lion hospital, Addis Ababa 9.3 (95% CI: 6.5, 12.1) [26]. The pooled burden of CKD among patients with diabetes was 18.2% (95% CI: 15.1, 21.3) (Figure 2).

**FIGURE 3:**
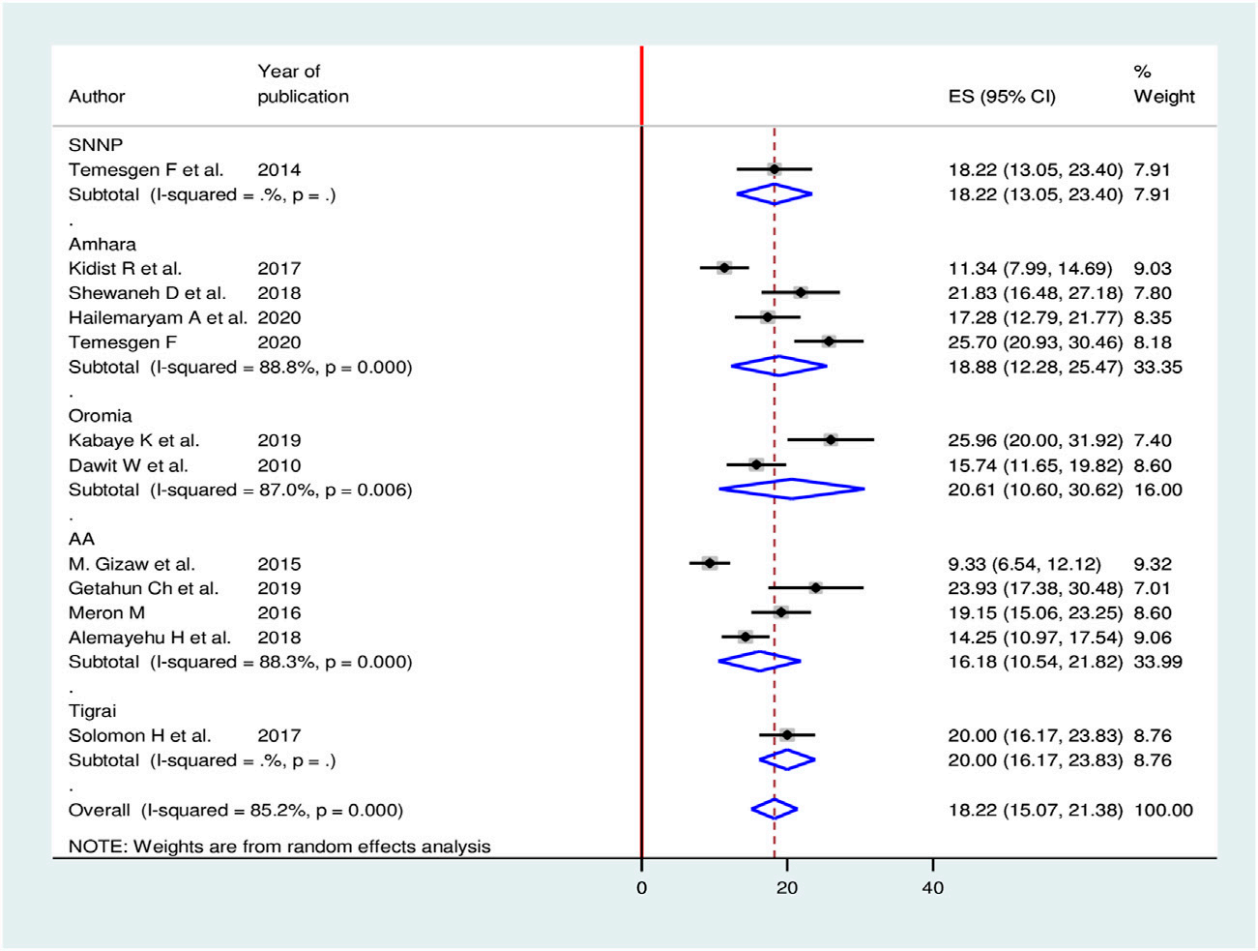
Sub group analysis based on the region for burden of CKD among patients with diabetes in Ethiopia, 2020.

Meta-regression was computed to see underlying sources of heterogeneity using sample size and year of publication, but none of them showed a statistically significant presence of heterogeneity (Table 2). Moreover, to minimize potential heterogeneity, subgroup analysis was conducted based on the region where the studies were conducted. The sub-group analysis result showed the highest burden in the Oromia region while the smallest was seen in Addis Ababa (Figure 3).

**TABLE 2:** Meta regression using sample size and year of publication to observe related heterogeneity on the burden of CKD among diabetic patients in Ethiopia, 2019.

Variables	Coefficients	*p*-value
Publication year	0.0344531	0.309
Sample size	0.0008549	0.058

**FIGURE 4:**
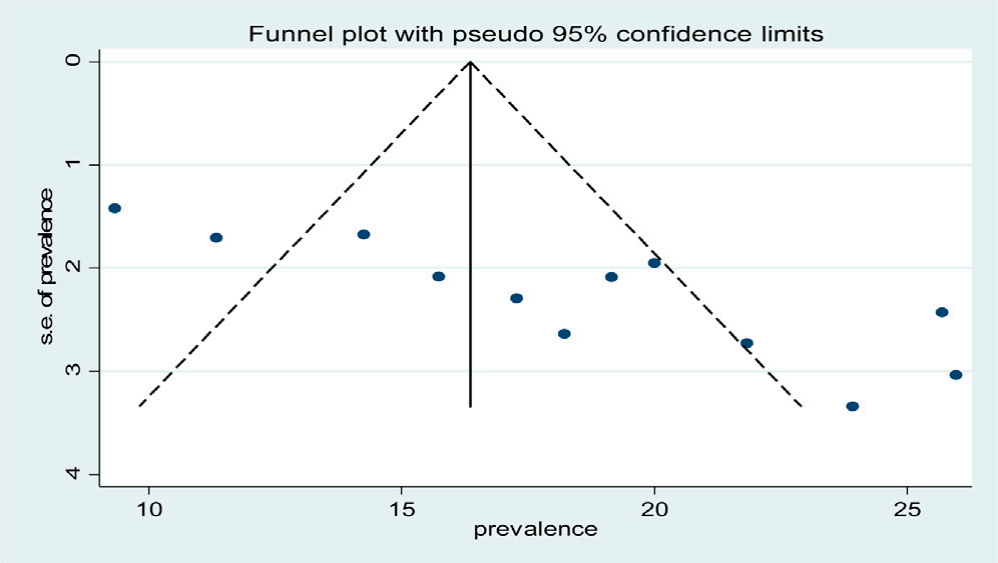
Funnel plot with 95% confidence limits of the pooled burden of CKD among patients with diabetes in Ethiopia, 2020.

To see for the presence of publication bias, a graphical funnel plot and Egger’s test at a 5% significance level were computed (Figure 4). The asymmetric funnel plot indicates the presence of publication bias. In addition, Egger’s test showed there was a statistically significant presence of publication bias (*p*-value = 0.041) but an insignificant presence of publication by using Begg’s test (*p*-value = 0.326).

**FIGURE 5:**
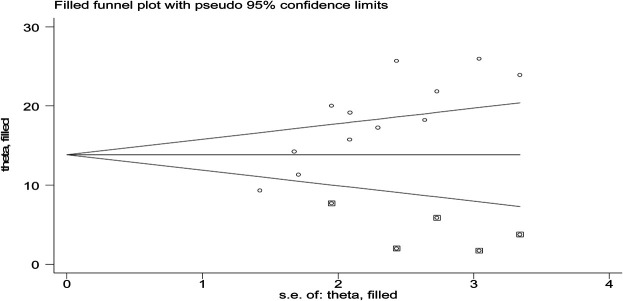
Result of trim and fill analysis for adjusting publication bias of the 12 studies, 2020.

To reduce and adjust the publication bias in the studies, the trim and fill analysis was performed for estimation of the number of missing studies that might exist. Trim and fill analysis is a nonparametric method for estimating the number of missing studies that might exist and it helps in reducing and adjusting publication bias in meta-analysis. In trim and fill analysis, five studies were imputed for missing studies and after adjustment for publication bias, the estimated pooled burden of CKD was 14.31 (95% CI: 10.95, 17.68) (Figure 5).

**FIGURE 6:**
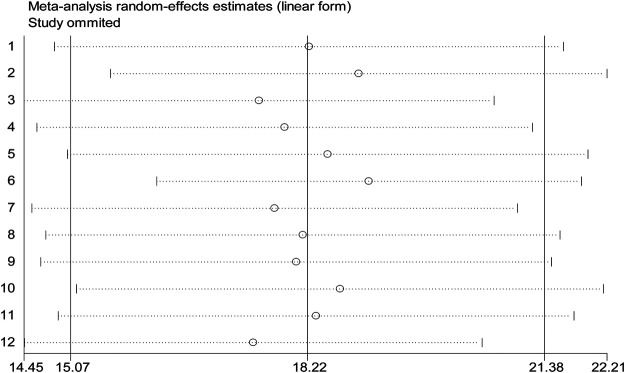
Sensitivity analysis for single study influence on the overall study of burden of CKD among patients with diabetes in Ethiopia, 2020.

Sensitivity analyses of the studies were done to test the effect of a single study on the pooled result of remaining studies using a random-effect model. We found no strong evidence for the influence of individual study on remaining studies (Figure 6).

### Determinants of Chronic Kidney Disease Among Patients With Diabetes in Ethiopia

#### Sex and Chronic Kidney Disease

**FIGURE 7:**
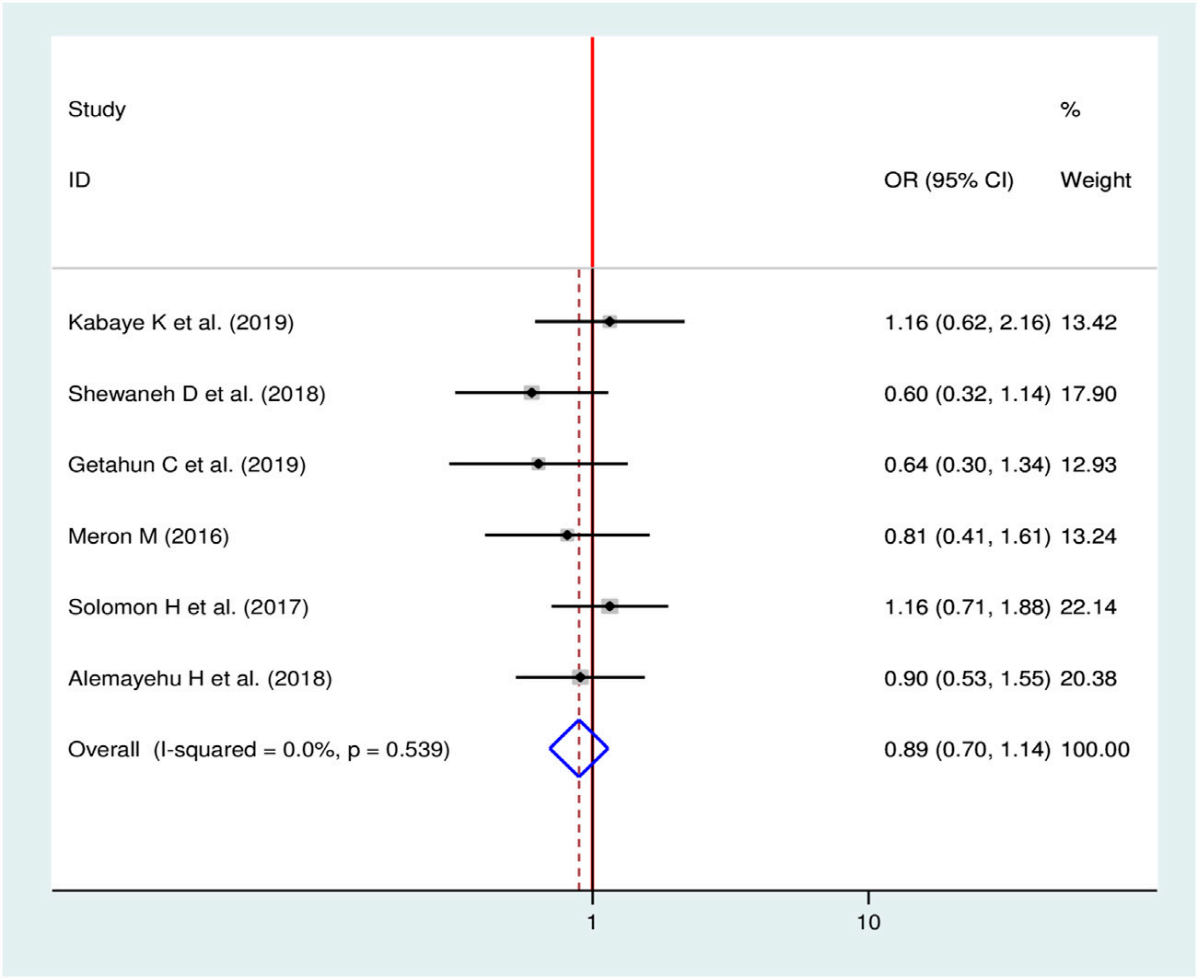
Forest plot for pooled effect sex on CKD among patients with diabetes in Ethiopia, 2020.

To see the effect of sex on CKD among diabetic patients, six studies were included in meta-analysis [11, 16–18, 24, 28]. The pooled result showed that, there was no a statistically significant association between sex of the patients and CKD among diabetic patients in Ethiopia (OR = 0.89, 95%, CI: 0.70, 1.14) (Figure 7).

#### Hypertension and Chronic Kidney Disease

**FIGURE 8:**
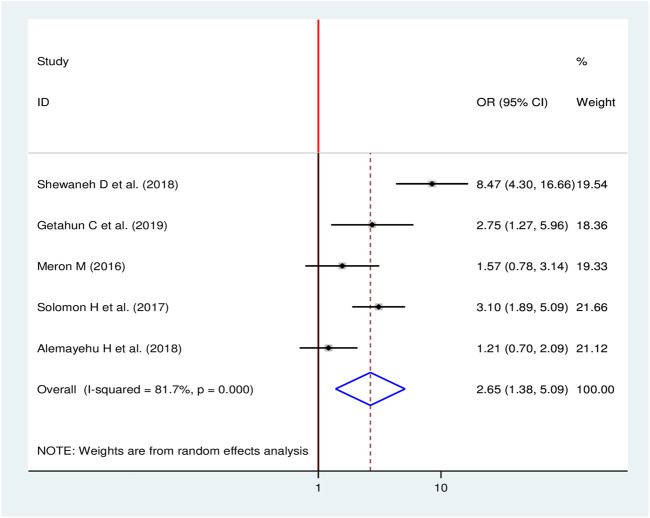
Forest plot for pooled effect hypertension on chronic kidney disease among diabetic in Ethiopia, 2020.

To observe the pooled effect of hypertension on CKD, five studies were selected in the final meta-analysis [11, 16, 18, 24, 28]. Since high heterogeneity was observed (I^2^ = 81.7, *p*-value < 0.001), a random-effects model was used to report the effect of hypertension on CKD. Three studies [16, 18, 28] showed the presence of a statistically significant association between hypertension and CKD, while two studies [11, 24] did not show a statistical significant association. The finding discovered that the odds of developing CKD were 2.65 times more likely among hypertensive patients than non-hypertensive patients (OR = 2.65, 95%, CI: 1.38, 5.09) (Figure 8).

#### Types of Diabetes Mellitus and Chronic Kidney Disease

**FIGURE 9:**
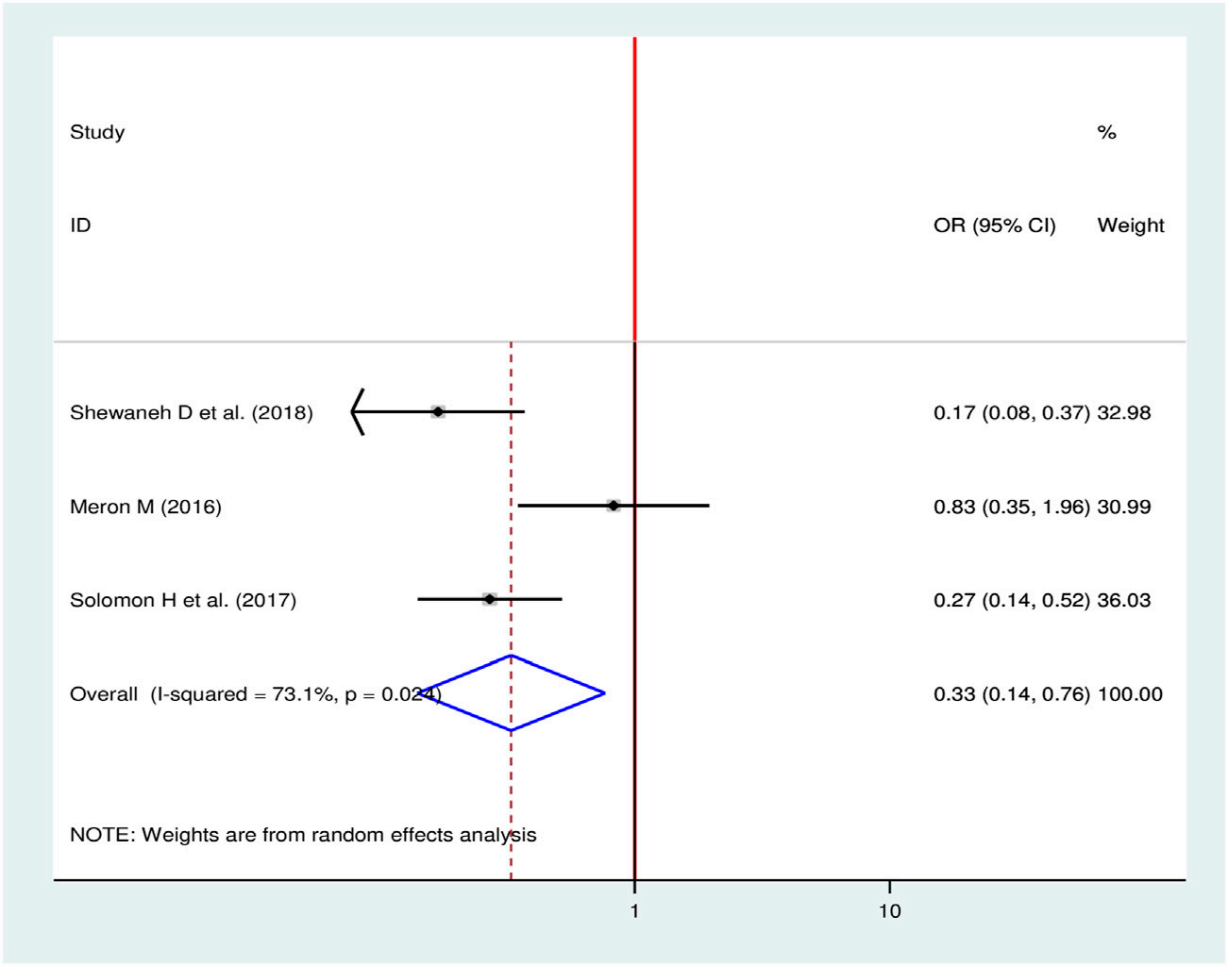
Forest plot for pooled effect of types of DM on CKD among patients with diabetes in Ethiopia, 2020.

To compute the effect of types of DM on CKD, three studies were selected for meta-analysis [16, 24, 28]. A random-effects model was used to estimate the pooled effect of types of DM on CKD (I^2^ = 73.1, *p*-value < 0.024). Two of the included studies showed a statistical significant association [16, 28] while one study did not indicate a statistical significant association between types of DM and CKD [24]. The pooled result of the analysis revealed type I DM decrease the odds of developing CKD by 67% as compared to type II DM (OR = 0.33, 95%, CI: 0.14–0.76) (Figure 9).

#### Family History of Kidney Disease and Chronic Kidney Disease

**FIGURE 10:**
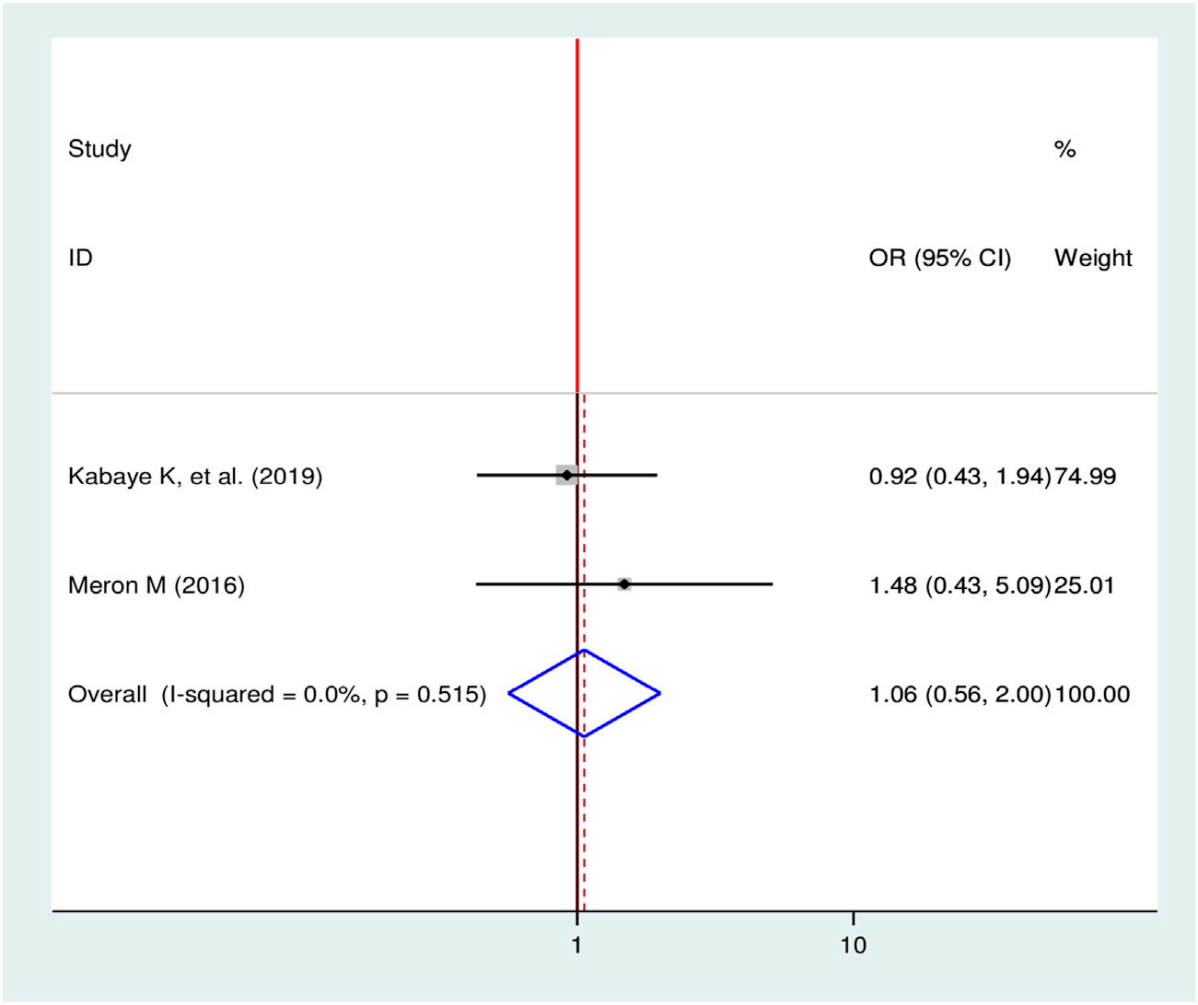
Forest plot for pooled effect of family history of kidney disease on CKD among patients with diabetes in Ethiopia, 2020.

Two studies were selected to observe the effect of family history of kidney disease on CKD among diabetic patients [17, 24]. Both studies showed no statistical significant association between family history of kidney disease and CKD (OR = 1.06, 95% CI: 0.56, 2.00) (Figure 10).

#### Body Mass Index and Chronic Kidney Disease

**FIGURE 11:**
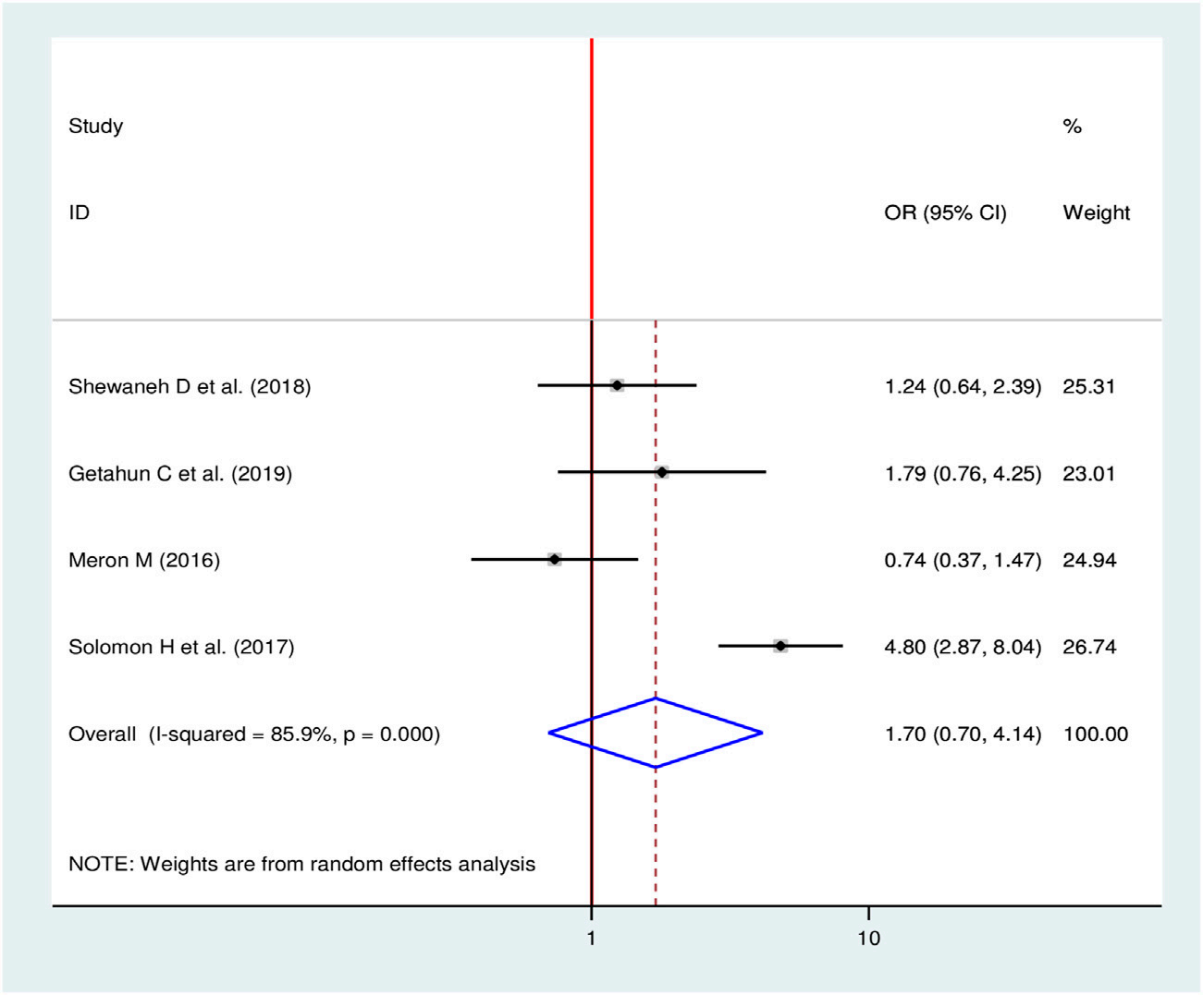
Forest plot for pooled effect of BMI on CKD among patients with diabetes in Ethiopia, 2020.

To identify the association between BMI and CKD, four studies were selected for meta-analysis [16, 18, 24, 28]. One study showed a statistical significant association [28] and three studies revealed no significant association between BMI and CKD. The pooled finding uncovered no significant association between BMI and CKD among diabetic patients (OR = 1.70, 95% CI: 0.70, 4.14) (Figure 11).

#### Duration of Diabetes Mellitus and Chronic Kidney Disease

**FIGURE 12:**
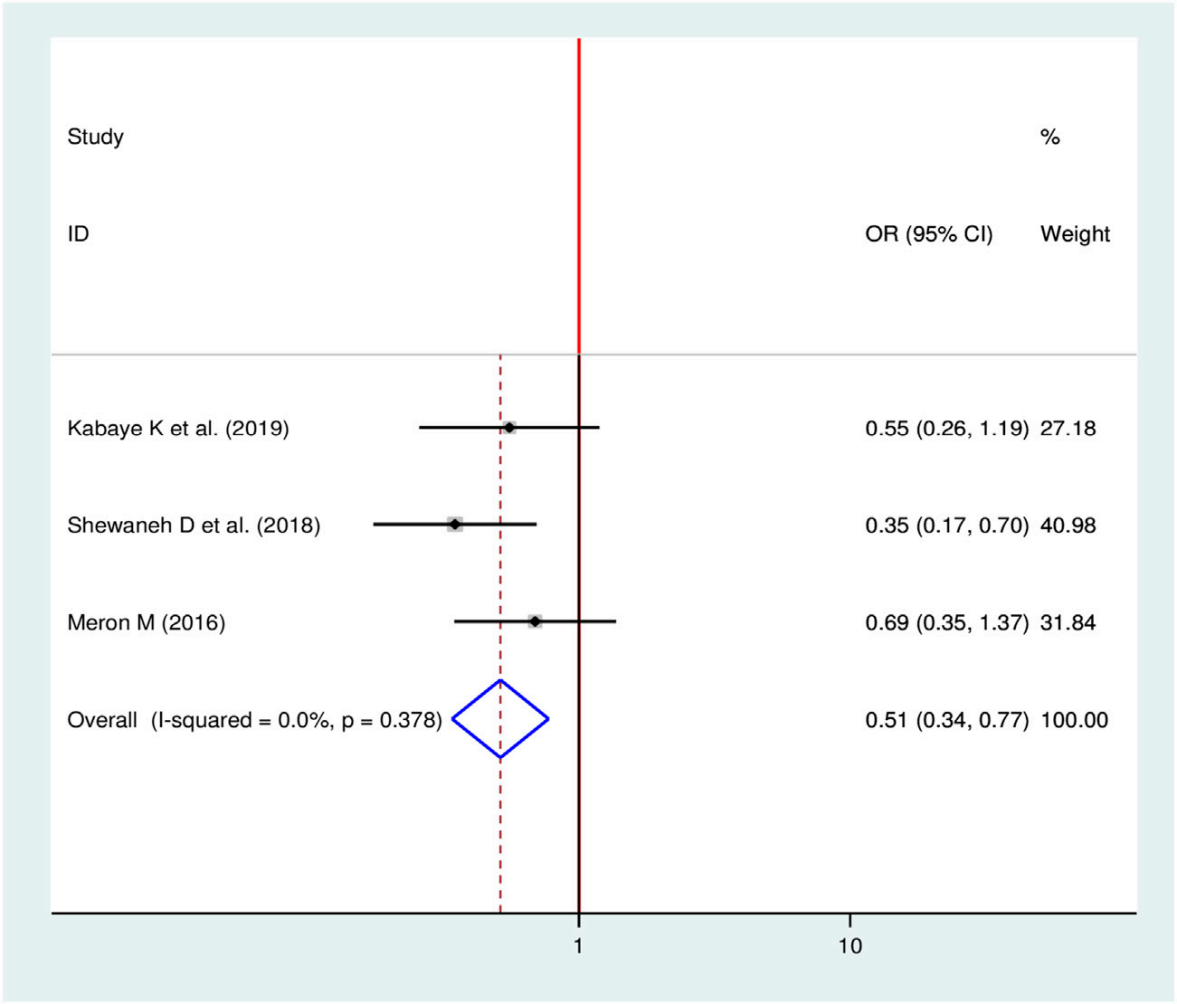
Forest plot for pooled effect of duration of patient stayed with DM on CKD among patients with diabetes in Ethiopia, 2020.

Three studies were identified to see the effect of duration of patients stayed with DM on the occurrence of CKD [16, 17, 24]. The pooled finding figures out that, the duration of the patients stayed with DM were significantly associated with the development of CKD among diabetic patients. Being diabetic patients for less than 10 years decreases the odds of developing chronic kidney disease by 49% as compared to diabetic patients who stayed with DM for ≥10 years (OR = 0.51, 95%, CI: 0.34–0.77) (Figure 12).

#### Alcohol Consumption and Chronic Kidney Disease

**FIGURE 13:**
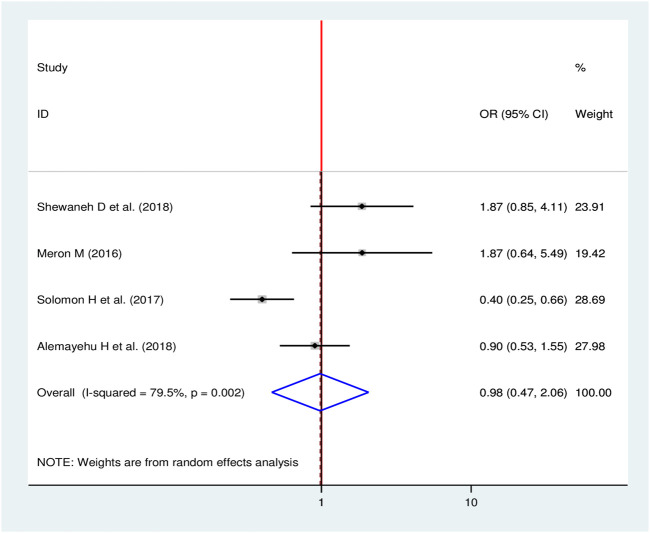
Forest plot for pooled effect of alcohol consumption on CKD among patients with diabetes in Ethiopia, 2020.

Four studies were included in the final meta-analysis to see the effect of alcohol consumption on CKD [11, 16, 24, 28] of which, a single study showed significance association between alcohol consumption and chronic kidney disease [28]. The pooled result showed that there was no significant effect of consuming alcohol on CKD (OR = 0.98, 95% CI: 0.47, 2.06) (Figure 13).

## Discussion

The burden of chronic non-communicable diseases (CNCDs) is increasing in Ethiopia. Different small scale studies are indicating the incremental pattern of CKD in diabetic patients in different regions of the country [16, 18, 28]. However, there is a paucity of representative evidence to show the burden and the determinants of CKD among diabetic patients in Ethiopia.

The finding indicated that the magnitude of CKD among diabetic patients in Ethiopia ranges from 9.3% (95% CI: 6.5–12.1) to 25.9% (95% CI: 20.0–31.9). The pooled magnitude of CKD was 18.2% (95% CI: 15.1–21.4) with significant heterogeneity across the studies. The result is relatively low when compared to the systematic review of diabetic nephropathy in Africa which ranged from 11 to 83.1% [14]. The observed difference might be due to the review includes studies from over 16 African countries.

Another analysis of systematic review on the burden of CKD among a general population and high-risk groups in Africa found the magnitude of CKD among diabetic patients ranging from 11% to 90% with a pooled prevalence of 24.7% (95% CI: 23.6–25.7) which was higher than the result of current meta-analysis [8]. The discrepancy could be due to the difference in the number of studies included in the analysis. In addition, the current findings was lower than the finding from China 35.5, 24.6% [29, 30], and Cape Town 20.7% [31]. Studies conducted in Greece and Thailand found the prevalence of CKD among diabetic patients was 34.7 [32] and 24.4% [33], respectively, which is higher than the current review. The discrepancy could be due to differences in the target population. The previous study was conducted among type 2 DM patients while our finding was included both type 1 and 2 DM patients.

However, the current finding is higher than reports from Nigeria (7.8%) [34], Malawi (1.4%) [35], Indonesia (4%) [36] and Sudan (13.3%) [37]. This might be due to the difference in study design, sample size, criteria of selection of the study participants, and study area. A study conducted in China revealed the prevalence of CKD among diabetic patients was 12.1% which lower than the current review. The discrepancy could be due to a higher population of China consumed nephrotoxic drugs or Chinese herbs which are assumed to have a preventive effects against CKD [38].

The odds of developing CKD for those who had hypertension were increased more than two folds when compared to CKD patients without a history of hypertension. This finding is in agreement with other results [8, 9, 14, 30, 35, 37]. The possible explanation for this could be the underachievement of blood pressure targets that could attribute to early end-organ damage and late presentation of medical care in patients with hypertension and diabetes mellitus [9, 14, 39]. This also pointed to the growing evidence for the existence of genetically determined and environmentally-induced factors responsible for the high risk of damage to the kidney, especially in countries like Ethiopia. Inadequate blood pressure control may explain the prevalence of CKD reviewed; however, poor renal function may also lead to an increased risk of blood pressure.

Types of diabetes mellitus were one of the prominent factors to contribute to the development of CKD among diabetic patients. Being a type I diabetes mellitus decrease the odds of developing CKD among diabetic patients. Evidence indicated that an increased burden of type II diabetes mellitus in developing countries including Ethiopia is closely linked to an increase in obesity. Because, developing countries are experiencing rapid demographic and epidemiological transitions which are characterized by a westernization of societies (plentiful foods, labor-saving machinery, and longer lifespan) [40]. There is also the pathophysiological confirmation that type II DM differs from type I to be significantly associated with CKD. Micro/macroalbuminuria may be present when type II DM is diagnosed, reflecting its long-lasting asymptomatic period and hypertension more commonly accompanies micro/macro albuminuria in type II DM [41]. It is obvious that metabolic disorders including type II DM and other cardiovascular diseases are closely related to aging. As type II DM increase among the aged population, the risk of developing CKD also increase among this population because there is a direct relationship between type II DM and CKD among the older age group.

The odds of developing CKD among diabetic patients was greatly reduced in diabetic patients who stayed with DM for less than 10 years when compared to those who stayed with the diseases for more than 10 years. This study goes in line with other study results [1, 10, 42]. This could be explained as an increased burden of type II DM in the population and improved survival of patients with type II DM patients. In another way, as the disease progress over time, *β*-cell function and insulin secretion decrease. This in turn facilitates for the advancement of CKD among the patients.

### Limitation of the Study

Even though the review will help as a paramount importance in contributing to providing recent evidence in Ethiopia, it has some limitations which need to be considered when used for further studies. The first and for most, relatively small sample size was used in almost all studies. Most of the studies included were cross-sectional with only one study case-control and retrospective cohort design each. All studies were health facility-based studies assessing diabetes mellitus patients on regular follow-up or admitted which could hinder the generalizability of the study leaving aside the diabetic patients left undiagnosed in large population. Even though, subgroup analysis was performed, a heterogeneous nature of the data was one of the limitations this study.

### Conclusion

In conclusion, the current review indicated that there is a high burden of CKD among patients with diabetes in Ethiopia. Determinants of CKD among patients with diabetes were presence of hypertension, type of diabetes mellitus, and duration of time the patient stayed after diagnosis. For better control of chronic kidney disease, integrated management of hypertension and DM should be designed with a special focus on chronic diabetic patients. A well-designed surveillance of high blood pressure and hyperglycemia is a better way to prevent and control the disease. Primary health care structures of the country should focus on early detection, proper screening, and management in order to reduce the effect of CKD on diabetic patients. Moreover, it is highly recommended to conduct a longitudinal study with representative samples both from health facility and community based at large to depict the clearly updated data to influence policymakers and program planners at national and international level.

## Supplementary Material

Supplementary File 1 PRISMA checklist.

## Abbreviations

BMI, body mass index; CI, confidence interval; CKD, chronic kidney disease; CNCD, chronic non communicable disease; DM, diabetes mellitus; OR, odd ratio; SNNP, southern nation nationalities and people; SSS, sub-saharan africa.
